# Selected Protective Mechanisms of Human Milk Against Intestinal Protozoal Infections in Infants

**DOI:** 10.3390/cimb47080674

**Published:** 2025-08-21

**Authors:** Joanna Wróblewska, Anna Długosz, Marcin Wróblewski, Jarosław Nuszkiewicz, Paweł Sutkowy, Alina Woźniak

**Affiliations:** 1 Department of Medical Biology and Biochemistry, Faculty of Medicine, Ludwik Rydygier Collegium Medicum in Bydgoszcz, Nicolaus Copernicus University in Toruń, 24 Karłowicza St., 85-092 Bydgoszcz, Poland; joanna.wroblewska@cm.umk.pl (J.W.); jnuszkiewicz@cm.umk.pl (J.N.); p.sutkowy@cm.umk.pl (P.S.); 2 Department of Food Industry Technology and Engineering, Faculty of Chemical Technology and Engineering, Bydgoszcz University of Science and Technology, 3 Seminaryjna St., 85-326 Bydgoszcz, Poland; anna.dlugosz@pbs.edu.pl

**Keywords:** amoebiasis, *Entamoeba histolytica*, giardiasis, human milk oligosaccharides, lactoferrin, *Giardia lamblia*, maternal milk, melatonin, mucins, neonates

## Abstract

Human milk, especially colostrum, is a biologically complex fluid with potent protective properties against gastrointestinal disturbances in infants. Among intestinal protozoa transmitted via the fecal–oral route, this review focuses on *Giardia lamblia* and *Entamoeba histolytica*, as the protective role of milk-derived factors against these parasites is the most extensively documented. Its protective effects result from a wide range of bioactive components, including mucins, lactoferrin, human milk oligosaccharides, melatonin, and secretory IgA, which support the integrity of the intestinal barrier, regulate immune responses, and inhibit the adhesion and activity of pathogens. The composition of human milk can be influenced by maternal factors such as nutritional status, stress, sleep quality, and physical activity, which may modulate its immunological potential. Dietary intake of micronutrients, fermentable fibers, and fermented foods also appears to play a role in shaping the milk’s protective properties. This review discusses the molecular mechanisms by which selected milk components contribute to the defense against protozoan infections in early life and considers how maternal health and lifestyle may affect the effectiveness of these protective mechanisms.

## 1. Introduction

The composition of human milk changes dynamically throughout lactation, adapting to the needs of the developing infant. Human milk typically contains 1–2% protein, 3–5% fat, and 6–8% carbohydrates. These concentrations vary between individuals, across different stages of lactation, and even throughout the day [1]. Its content also depends on gestational age, as mothers who deliver prematurely produce milk with higher concentrations of proteins (including lactoferrin) and higher levels of human milk oligosaccharides (HMOs) [2]. The multifunctional molecularly active bioactive molecules present in milk not only support neonatal development but also effectively protect against infections and inflammatory conditions. The highest concentrations of these components are found in colostrum, the first form of milk produced during the initial 5 days postpartum [3,4]. Colostrum is rich in proteins, immunoglobulins, and growth factors, and its primary function is to protect the newborn from infections and to support intestinal maturation [4]. The milk fat globule membrane in its natural form is a tri-layered structure composed of various biological components. It consists primarily of polar lipids and proteins, which together account for over 90% of its dry mass. The lipid fraction includes phospholipids such as phosphatidylcholine (27.4–35.0%) and phosphatidylethanolamine (30.0–36.4%), sphingomyelin (17.3–29.8%), and cholesterol, which is the predominant sterol component [5]. Among the proteins that constitute the milk fat globule membrane, particular attention is drawn to mucin and lactoferrin. These bioactive glycoproteins derived from maternal milk serve essential protective functions in the infant’s gastrointestinal tract. Mucin, specifically MUC1, due to its structure rich in sialic acid and its high resistance to digestive enzymes, can pass through the newborn’s gastrointestinal system largely intact [4]. Its presence has been confirmed in the feces of breastfed infants, indicating that it retains its biological properties within the intestinal lumen [6]. Mucins present in the milk fat globule act protectively by blocking infections caused by viruses such as HIV and rotaviruses, bacteria such as *Salmonella spp.*, and infestations caused by protozoa [3,7]. The second most important glycoprotein, lactoferrin, also exhibits antimicrobial activity, acting against viruses, bacteria, fungi, and protozoa [2,3]. It binds iron ions, limiting their availability to pathogenic bacteria, while simultaneously supporting the development and maturation of the infant’s immune system [2]. Human milk contains over 200 structurally diverse oligosaccharides, known as HMOs, which are unconjugated complex glycans. Their total concentrations can reach 20–25 g/L in colostrum and decrease to 5–20 g/L in mature milk. Only a small number of specific structures, such as 2′-fucosyllactose and lacto-*N*-neotetraose, have been added to some modern infant formulas. These synthetic HMOs are present at relatively low concentrations and do not reflect the structural complexity of those naturally present in human milk [1,8]. These synthetic HMOs are present at relatively low concentrations and do not reflect the structural complexity of those naturally present in human milk. According to the European Food Safety Authority, the maximum permitted levels of individual synthetic HMOs in infant formulas range from 0.2 to 2.4 g/L [9]. All HMOs contain a lactose molecule at their reducing end, which may be fucosylated or sialylated. The dominant type of HMO in maternal milk depends on the stage of lactation. HMOs are not digested by the human body. Over 90% of them can be detected in the infant’s stool, while less than 1% is excreted in the urine. Remaining in the intestinal lumen, they exert their primary function of protecting against enteric pathogens [2]. Additionally, the concentration of metabolic hormones in breast milk may significantly influence infant body weight, fat tissue composition. However, current research findings are inconsistent, indicating a need for further studies in this area [10]. Of particular interest is melatonin, also present in maternal milk, which plays a crucial role in regulating the circadian rhythm. Its concentration in human milk follows a clear circadian pattern, peaking at night and disappearing during the day. This is particularly important for newborns, who do not endogenously produce melatonin during the first months of life. Melatonin exhibits antioxidant, anti-inflammatory, and immunomodulatory properties, and may also influence the balance of the gut microbiota and alleviate metabolic disorders [10].

Together, these bioactive components form a key element of the infant’s innate immune defense, playing a role far beyond simple nutritional functions and potentially protecting against the consequences of protozoan infestations. It is widely accepted that exclusive breastfeeding protects infants against diarrhea. Human milk is an important source of bacteria colonizing the infant gut, containing hundreds of microbial species, with breastfed infants potentially ingesting up to 800,000 microorganisms daily, primarily from genera such as *Streptococcus*, *Staphylococcus*, *Micrococcus*, *Lactococcus*, *Lactobacillus*, and *Bifidobacterium* [11,12,13]. This supports the development of a diverse gut microbiota, which can protect against infections, including those caused by *Giardia lamblia* and *Entamoeba histolytica* [14,15,16]. Intestinal protozoa transmitted via the fecal–oral route and responsible for gastrointestinal infections include *G. lamblia*, *E. histolytica*, *Cryptosporidium* spp., *Blastocystis* spp., *Cyclospora cayetanensis*, and *Cystoisospora belli* [17]. Newborns, and especially preterm infants, are particularly vulnerable to gastrointestinal infections due to the immaturity of their digestive and immune systems. Increased intestinal permeability and the limited production of innate and adaptive immune effectors contribute to a higher susceptibility to enteric pathogens during early life [18,19]. Antibodies, especially secretory IgA (sIgA) present in breast milk, play an important role in protection against protozoan infestation. High levels of IgA antibodies targeting the Gal/GalNAc lectin in maternal milk have been associated with a reduced frequency of *E. histolytica* infestation in infants, and the presence of these antibodies in infant stool correlates with a longer period free from subsequent infestation [14]. Human milk also contains other antimicrobial components, such as lactoferrin, oligosaccharides, leukocytes, and cytokines, which, together with antibodies, support local immunity in the gastrointestinal tract and may reduce both the frequency and severity of protozoan infestations [20,21].

The aim of this paper is to discuss the biological significance of selected components of human milk, in protecting newborns and infants against protozoan infestation of the gastrointestinal tract, with particular focus on *G. lamblia* and *E. histolytica*, for which the protective role of milk-derived factors is the most extensively documented. The paper focuses on the mechanisms of action of bioactive components such as mucins, lactoferrin, melatonin, and HMOs in maintaining intestinal epithelial integrity, supporting immune system maturation, and limiting the presence of parasites in the infant gut. The paper also discusses the influence of maternal nutritional status and lifestyle on the immunological composition of human milk, with particular emphasis on components that protect infants against parasitic infections.

## 2. Intestinal Protozoan Infections in Children: Pathogenesis, Clinical Manifestations, and Impact on the Intestinal Barrier

Giardiasis, caused by the protozoan *G. lamblia* (also known as *G. duodenalis*, *G. intestinalis*, or *Lamblia intestinalis*), is one of the most commonly diagnosed parasitic diseases of the gastrointestinal tract worldwide [22]. In immunocompetent individuals, most cases are self-limiting and resolve spontaneously within a few weeks of exposure. The disease primarily manifests through gastrointestinal symptoms such as abdominal pain, bloating, diarrhea, and nausea. Chronic giardiasis is typically associated with steatorrhea and deficiencies in fat-soluble vitamins (e.g., A, K) as well as water-soluble ones (e.g., B_12_) [7]. Vitamin A malabsorption during *G. lamblia* infection has been linked to small intestinal mucosal damage and microvilli dysfunction. In addition, hepatic vitamin A stores are mobilized in an attempt to maintain serum retinol levels [23]. Several mechanisms have also been proposed to explain impaired vitamin K absorption. These include the formation of a physical barrier by the parasite that limits nutrient uptake, pancreatic insufficiency affecting digestion, and altered bile salt metabolism in the small intestine [24]. In the case of vitamin B_12_, malabsorption has been reported in giardiasis. Proposed mechanisms include direct competition between the parasite and the host for available vitamin B_12_, and the development of bacterial overgrowth secondary to infection [25]. Persistent malabsorption in children with chronic giardiasis may lead to growth retardation or signs of undernutrition, particularly in settings where dietary intake is already limited [7,26,27]. In pediatric populations, infection is often chronic and asymptomatic; however, experimental studies indicate that even asymptomatic giardiasis may impair growth, alter the gut microbiota, and disrupt lipid and bile acid metabolism. Infection during infancy may have long-term consequences for a child’s physical development [16,26,27]. In more severe cases, active giardiasis can lead to secondary lactose intolerance, which may persist for several months after the infection has resolved [28]. There is also evidence suggesting a potential link between giardiasis and increased susceptibility to food allergies. Children infected with *G. lamblia* may present with elevated total IgE levels and specific IgE antibodies against food antigens [29]. Breastfed infants are significantly less likely to develop chronic diarrhea due to *G. lamblia* infection, and the intensity of infection tends to be milder compared to formula-fed infants [21].

Transmission occurs via the fecal–oral route, typically through the ingestion of food or water contaminated with quadrinucleate, oval-shaped cysts. As few as 10–25 cysts may be sufficient to colonize the small intestine. In the intestinal lumen, cysts transform into vegetative forms, known as trophozoites, which are pear-shaped with two nuclei and four pairs of flagella. Trophozoites primarily colonize the duodenum, and less frequently, the jejunum and ileum [7,30].

During giardiasis, the intestinal epithelial barrier is significantly disrupted by the coordinated action of *G. lamblia* trophozoites and their secreted cysteine proteases, which target tight and adherens junctions. Zonula occludens-1 (ZO-1) undergoes flocculation and relocalization; occludin and claudins (particularly claudin-1 and claudin-4) are both degraded and displaced from their normal junctional sites; junctional adhesion molecule 1 is partially cleaved; β-catenin exhibits similar relocalization and partial degradation; while E-cadherin is redistributed within epithelial cells and efficiently cleaved by recombinant proteases in vitro [31]. Concurrently, the actin cytoskeleton is reorganized, destabilizing epithelial cells and reducing adhesion, and apoptotic pathways are activated in enterocytes, primarily through caspase-3 activation. Loss of epithelial cells further weakens the continuity and tightness of the intestinal layer. This process is intensified by local inflammation with increased production of proinflammatory cytokines such as tumor necrosis factor alpha (TNF-α) and interleukin 6 (IL-6), which aggravate barrier dysfunction. Infection also affects the mucus layer, resulting in its thinning and changes in the number and function of goblet cells, leading to reduced mucin production. These combined disturbances increase intestinal permeability, facilitating the translocation of antigens and microorganisms into deeper layers of the intestinal wall, intensifying inflammatory responses and clinical symptoms [7]. Infections caused by *G. lamblia*, like other gastrointestinal diseases such as inflammatory bowel disease or irritable bowel syndrome, are closely associated with alterations in the composition of gut microbiota and increased intestinal permeability [32].

Amebiasis, caused by *E. histolytica*, is one of the most serious parasitic diseases of the gastrointestinal tract in tropical and subtropical regions, particularly affecting malnourished children [33,34,35]. A prospective cohort study by Mondal et al. [36] demonstrated that the incidence of *E. histolytica*-associated diarrhea was more than three times higher in malnourished children (5.45 per 100 child-years) compared to well-nourished children (1.76 per 100 child-years, *p* = 0.004). Although symptomatic amebiasis is rare in neonates, it can cause severe complications such as colitis or symptoms resembling necrotizing enterocolitis, with some cases associated with the early postnatal use of water or sugar solutions in place of breast milk [34,37]. Depending on the affected organs, the clinical presentation of amebiasis is classified as intestinal or extraintestinal [38]. In children, infection is characterized by watery or mucoid diarrhea, often with blood, vomiting, feeding refusal, abdominal distension, and altered bowel sound [33,37]. In infants and neonates, the disease may have a much more severe course, and the prognosis is usually worse than in older children [39]. Additionally, breastfeeding has been shown to reduce the risk of *E. histolytica* infection compared to formula feeding, which may be explained by lower exposure to contaminated water used in formula preparation, as well as the presence of natural amoebicidal compounds in breast milk [40]. Invasive amebiasis develops in only one in five infected individuals [41]. In severe cases, it can lead to life-threatening multi-organ complications, including abscess formation in the liver, lungs, brain, and spleen, with central nervous system involvement being associated with a mortality rate of up to 90% [39]. The mechanisms underlying the transition from asymptomatic to invasive disease remain unclear, but the gut microbiota is believed to play a key role. The presence of *E. histolytica* alters the composition of the host’s gut microbiome, with a reduction in beneficial bacteria such as *Bacteroides* spp. and *Lactobacillus* spp., leading to dysbiosis [41]. Reduced microbial diversity is also considered a risk factor for developing amebiasis [35].

The trophozoite of *E. histolytica* resides in the lumen of the large intestine, where it multiplies and transforms into cysts, the infective form. Cysts are excreted in feces and may be ingested by a new host through contaminated food or water. The parasite encysts in the large intestine, and the cysts are eliminated in stool [38]. Trophozoites can invade the mucosal layer of the large intestine and spread via the bloodstream to cause extraintestinal manifestations. A critical step in the pathogenesis of intestinal invasion by *E. histolytica* is the disruption of the protective mucus layer. The parasite first adheres to the mucus using, among other factors, the Gal/GalNAc lectin, and then degrades the mucus. This degradation occurs through two mechanisms: by stimulating mucin secretion, leading to goblet cell depletion, and by directly degrading mucins using cysteine proteases and glycosidases [41]. A key mechanism of epithelial damage involves the EhCPADH112 protein complex, which binds to tight junction proteins such as occludin, claudin-1, ZO-1, and zonula occludens-2, causing their degradation and thereby compromising the integrity of the intestinal barrier [42].

## 3. Mucins as a Component of Innate Immunity in Infections with *Giardia lamblia* and *Entamoeba histolytica*

The gastrointestinal mucosa represents the primary entry point for many intestinal pathogens. These microorganisms must overcome natural innate immune barriers, such as the mucus layer covering the intestinal epithelium. In this way, the mucus layer limits the contact between pathogens and their antigens with the epithelial surface, acting as a form of non-immune exclusion [7]. Mucins, high-molecular-weight glycoproteins, play a crucial role in this mechanism. Mucins are divided into two main groups based on their structure and location: transmembrane mucins, such as MUC1, MUC3, MUC4, MUC12, MUC13, MUC16, and MUC17, which are anchored in the cell membrane; and secreted mucins, which are further divided into gel-forming mucins, such as MUC2, MUC5AC, MUC5B, and MUC6, and non-gel-forming mucins, such as MUC7 and MUC20 [43].The best-studied mucin with documented protective properties against *G. lamblia* and *E. histolytica* invasion is MUC2 [32,35].

Mucins such as MUC2 undergo extensive *O*-glycosylation, in which typical *O*-glycan core structures, including core 1, core 2, core 3, and core 4, are attached to serine and threonine residues, and are further elongated and modified by fucosylation and sialylation. This type of glycosylation plays a key role in defining the biochemical properties of mucins, influencing both the organization and function of the mucus layer, as well as the interaction between mucins and microorganisms [32]. Mucins constitute the first line of defense against many intestinal pathogens, including *G. lamblia* and *E. histolytica* [7,30]. *O*-glycosylation may protect mucins from degradation by host enzymes and bacterial enzymes, and the glycans themselves serve as binding sites for commensal microbiota, supporting its maintenance and limiting microbial penetration through the epithelium [32].

The presence of mucins may hinder the adhesion of *G. lamblia* trophozoites to the epithelial surface, likely through electrostatic repulsion between the negatively charged mucins and the parasite surface [44]. However, not all components of mucus exert protective effects. Low-density, protein-rich mucus fractions devoid of mucins, isolated from the human or rabbit duodenum and ileum, have been shown to promote the adhesion and survival of *G. lamblia* trophozoites in vitro in a dose-dependent manner [45]. Moreover, these fractions protected the parasites from the antimicrobial activity of human milk, most likely by limiting lipase activity or binding lipolytic products such as fatty acids [46]. Pathogens may also exploit O-glycan structures present on mucins to initiate infection, modify them, or degrade them to obtain nutrients [32]. Although intestinal protozoa vary in their capacity to degrade mucins, *G. lamblia* has been found to possess enzymatic activity of β-N-acetylglucosaminidase and detectable levels of β-N-acetylgalactosaminidase, suggesting an ability to effectively degrade mucus components [7]. *Giardia* infection impacts the structure and function of the mucus layer both indirectly, by inducing dysbiosis of the gut microbiota, and directly, via cysteine proteases secreted by the parasite. Although these protozoa do not degrade mucins as effectively as some other enteric pathogens, their presence leads to alterations in mucin glycosylation, particularly MUC2; changes in the expression of mucin-encoding genes; and may result in thinning of the mucus layer, especially in the colon [32,47].

*E. histolytica* possesses a surface lectin that recognizes residues of galactose and N-acetylgalactosamine (Gal/GalNAc), which plays a key role in adhesion to host cells and is associated with the parasite’s virulence. This lectin also shows affinity for colonic mucins, suggesting that mucins may serve a protective function by hindering amoebic attachment to the epithelium, thereby limiting their cytolytic activity and movement within the mucus layer [48]. To cause infection, *E. histolytica* must overcome the mucus barrier by degrading the mucin layer and attaching to the intestinal epithelial cells. The parasite produces a range of cysteine proteases, among which the best-characterized is *E. histolytica* cysteine protease 5. In vitro studies have shown that this enzyme cleaves the cysteine-rich C-terminal domain of the MUC2 mucin. Cysteine proteases are essential for overcoming the protective function of mucins, although they do not directly determine the cytotoxicity of the parasite [35]. After breaching the mucus barrier and adhering to the epithelium, the parasite also utilizes the cysteine protease EhCP112, which degrades tight junction proteins, particularly claudin-1 and claudin-2. This leads to the loosening of intercellular junctions and a decrease in transepithelial electrical resistance [49]. Colonization of the mucin layer by *E. histolytica* triggers an inflammatory response. One of the key virulence factors of the parasite is prostaglandin E_2_, produced by *E. histolytica*, which induces a strong inflammatory state, causing nonspecific tissue damage and facilitating the penetration of trophozoites into the colonic mucosal layer [50].

## 4. Maternal Antibodies and Breastfeeding in the Defense Against Parasitic Protozoan Infections in Infancy

Human breast milk has demonstrated in vivo protective effects against protozoan infections in infants. Both epidemiological and experimental studies confirm that breastfeeding reduces the risk of infections caused by *G. lamblia* and *E. histolytica* [51,52,53,54]. Among the components of breast milk, secretory immunoglobulin A (sIgA) is considered particularly important in preventing intestinal parasitic infections in infants. sIgA is regarded as the first line of specific defense against natural infections across the extensive surfaces of mucosal tissues. Mucins and sIgA act together to capture microorganisms within the mucus layer, limiting their access to the intestinal epithelium or blocking their adhesion, thereby facilitating their removal from the body through intestinal peristalsis [55]. Fecal IgA antibodies directed against the carbohydrate recognition domain (CRD) of the *E. histolytica* Gal/GalNAc lectin have been associated with protection from subsequent intestinal amebiasis in children [55]. Human sIgA has also been shown to inhibit the enzymatic activity of a 70 kDa E. histolytica cysteine protease [56], and to recognize a 115 kDa surface antigen [57]. A similar protective role of sIgA has been observed in the context of *G. lamblia* infections. Secretory IgA present in breast milk has been shown to recognize several immunodominant *Giardia* antigens, including α-1 giardin, ornithine carbamoyl transferase, arginine deiminase, α-enolase, and variant-specific surface proteins (VSPs), with the latter exhibiting particularly strong reactivity [58]. Table 1 summarizes key findings from studies evaluating the protective role of breastfeeding, with particular emphasis on the antibodies shown to confer protection against *E. histolytica* and *G. lamblia* infections in infants.

## 5. Infections with *E. histolytica* and *G. lamblia* and the Potential Role of Human Milk Oligosaccharides in Infant Protective Mechanisms

Breastfed infants are traditionally thought to have a lower risk of diarrhea due to the presence of antibodies in human milk; however, oligosaccharides, particularly the content of 2′-fucosylated oligosaccharides in human milk, may also play a significant role in this protection [61]. HMOs are the third most abundant solid component of milk and the second most abundant complex sugar in human milk, after lactose. Although HMOs constitute only about 1% of its composition, they play an important biological role by supporting the development of microbiota characteristic of infancy that are capable of utilizing milk components, and their fucosylated structures, resembling glycans present on the surface of intestinal epithelial cells, may mimic their function and act as molecular decoys for pathogens, hindering their contact with the intestinal epithelium [62,63]. These compounds are not digested by the infant’s intestinal mucosa [62]. HMOs are composed of five basic monosaccharides: glucose, N-acetylglucosamine, galactose (Gal), fucose, and sialic acid [40,62,63].

The lectin of *E. histolytica* recognizes sugars such as Gal, GalNAc, and lactose, and some of these structures are also found in HMOs [40]. Additionally, the lectin participates in the killing and phagocytosis of host intestinal epithelial cells and is considered one of the main virulence factors of the parasite [8]. In vitro studies have shown that Gal, GalNAc, and lactose reduce the ability of *E. histolytica* trophozoites to adhere to host cells and decrease the cell damage they cause [40,64]. However, these sugars are usually digested and absorbed in the small intestine, which is why they are rarely present in the large intestine, the primary site of parasite colonization. In contrast to simple sugars such as galactose or lactose, HMOs and galactooligosaccharides are resistant to digestion in the small intestine and reach the large intestine unchanged, where they can interact with the microbiota and potentially affect pathogens such as *E. histolytica*. It has been demonstrated that HMOs significantly reduce the ability of *E. histolytica* to adhere to intestinal epithelial cells and decrease its cytotoxic activity toward human intestinal HT-29 cells. Under in vitro conditions, the use of HMOs at concentrations corresponding to their natural levels in human milk (10 g/L) caused detachment of more than 80% of the parasite’s trophozoites within the first thirty minutes. HMOs are not toxic to the parasite but prevent its stable adhesion to cells, suggesting a protective role by interfering with the adhesion mechanism. The human milk oligosaccharide 2′-fucosyllactose significantly reduces the cytotoxicity induced by *E. histolytica* against human intestinal epithelial cells. The protective effect was specific to 2′-fucosyllactose and was not observed with non-fucosylated analogs, indicating that the presence of the fucose residue in the HMO molecule is essential for inhibiting the parasite’s cytotoxicity [40].

To date, no peer-reviewed experimental studies have directly evaluated the effect of HMOs on *G. lamblia*. Available evidence is limited to preliminary, non–peer-reviewed findings, which should be interpreted with caution until confirmed by further research. These preliminary data suggested no effect of pooled HMOs on parasite proliferation and only a very slight, statistically significant reduction in adhesion to epithelial cells with 6′-sialyllactose (less than 3%) [65].

## 6. The Role of Lactoferrin in Limiting Intestinal Protozoan Infections

Lactoferrin (LF) is a multifunctional protein classified as a non-heme iron-binding glycoprotein belonging to the transferrin family; it is recognized as an antimicrobial peptide. The key role of lactoferrin lies in iron regulation. It exists in two forms: iron-bound (holo-lactoferrin) and iron-free (apo-lactoferrin). LF binds iron with high affinity in a reversible manner, which stabilizes its molecular structure and increases its resistance to enzymatic degradation and high temperatures. LF is capable of retaining iron in low pH environments, typical of the intestine, thereby supporting its absorption through a specific receptor located in intestinal epithelial cells. Once inside the cell, iron is reduced from Fe^3+^ to Fe^2+^, enabling its further metabolism. It is worth noting that Fe^2+^ is a more reactive form, capable of generating reactive oxygen species (ROS), which may lead to oxidative stress. Due to its chelating properties, LF reduces the availability of free iron, thereby limiting the formation of ROS and protecting cells from oxidative damage [66]. LF is the most abundant protein in the whey fraction of human milk. Its concentration varies depending on the stage of lactation. The highest lactoferrin concentration is observed in colostrum during the first 5 days postpartum, reaching approximately 7 g/L. It exceeds 5 g/L in early milk (<28 days after birth) and then significantly decreasing to 2–3 g/L in mature milk (≥28 days) [67]. However, during extended lactation, beyond 12 months, LF concentration increases again [68]. The breakdown of LF into active peptides such as lactoferricin (LFcin) and lactoferrampin (LFampin) during digestion may offer health benefits, as these peptides exhibit stronger antimicrobial activity than the native protein. The degree of LF degradation depends not only on the level of glycosylation and iron saturation, but also on the method of administration, gastric pH, and the type and activity of digestive enzymes, which differ significantly between infants and adults [69].

Recent reviews have emphasized the importance of LF as a multifunctional protective factor in human milk, highlighting its anti-protozoal activity against *G. lamblia* and *E. histolytica* [70]. LF and its derived peptides, such as LFcin and LFampin, exhibit strong anti-protozoal activity against *G. lamblia* and may function as components of non-immunological mucosal defense, acting directly with cytotoxic effects on intestinal parasites. [71]. This efficacy has been confirmed in in vitro studies, which demonstrated that both the full bovine LF molecule and its synthetic fragments, including LFcin17–30, LFampin265–284, and the hybrid peptide LFchimera, significantly reduce the viability and growth capacity of parasite trophozoites [72]. These peptides have been shown to be internalized into trophozoites through endocytosis. Once inside the cell, they induce membrane damage, pore formation, cytoplasmic disorganization, cytoskeletal rearrangement, and the appearance of programmed cell death markers [72]. They bind to the specific surface receptor GlLRP, which is involved in receptor-mediated endocytosis. This interaction may disrupt the normal course of encystation, leading to the formation of cyst-like structures that lack true dormancy properties. These forms are unstable and unable to survive in aquatic environments, significantly limiting the parasite’s ability to transmit [69]. The effectiveness of LF against *Giardia* spp. was also confirmed in a study conducted by Ochoa et al. [73], in which children were supplemented with bovine LF. Children in the LF group showed a lower prevalence of *Giardia* spp., a shorter duration of parasite carriage, and fewer cases of chronic infection compared to those who received a placebo. Additionally, children supplemented with LF achieved better growth indicators, which may suggest a beneficial effect of LF on physical development by limiting parasitic colonization and improving nutrient absorption.

LF exhibits strong anti-amoebic activity, improves health status without side effects, and may serve as an effective drug or an adjuvant to metronidazole therapy in the treatment of amoebic liver abscess [74]. *E. histolytica* has a high iron requirement, and limiting iron availability can inhibit its growth. In the environment of the large intestine, amoebae can obtain iron from host cells, such as shedding epithelial cells or erythrocytes during tissue destruction. However, under iron-restricted conditions, they do not exhibit invasive characteristics, suggesting that LF, by sequestering iron, may reduce their virulence and ability to colonize [75]. In the study by León-Sicairos et al. [57], the effects of milk, its fractions, and purified proteins (including lactoferrin, sIgA, and lysozyme) on the viability of protozoa suspended in BI-S-33 medium were evaluated, testing various concentrations and incubation times, both in the presence and absence of iron. Cell viability was assessed microscopically and by flow cytometry. The authors demonstrated that fresh human milk exhibited strong amoebicidal activity after just 1 h of incubation at 37 °C with gentle mixing by inversion, reducing trophozoite viability in a concentration-dependent manner (from 86% at 5% milk to 62% at 20%). Moreover, bovine milk showed weaker activity compared to human milk. Particular attention was given to lactoferrin, especially its apo form, which exhibited the strongest amoebicidal effect among all tested milk proteins. Apo-lactoferrin bound to the amoebic membrane, causing cell rounding, lipid disruption, and eventual cell death. Although data on fecal lactoferrin concentrations during intestinal amoebiasis were not available, the authors hypothesized that its levels likely increase due to neutrophil degranulation. This suggests that LF may also exert its activity against *E. histolytica* trophozoites in vivo.

## 7. Melatonin as a Natural Guardian in Early Life and Parasitic Defense

Melatonin is a small, lipophilic neurohormone that is mainly synthesized at night by the pineal gland and released into the bloodstream. In addition to its role in circadian rhythm regulation, melatonin and its metabolites (e.g., 6-hydroxymelatonin) exhibit strong antioxidant properties. Melatonin acts both as a direct antioxidant and indirectly by stimulating the activity of antioxidant enzymes such as superoxide dismutase (SOD) and glutathione peroxidase [76]. The protective properties of melatonin in human breast milk are also related to its anti-inflammatory and immunomodulatory effects. In newborns who do not yet produce endogenous melatonin, breast milk is their only source of this neurohormone. Its presence in colostrum and mature milk may therefore play a role in protecting against oxidative stress in early life [77]. Melatonin present in human breast milk exhibits a clear circadian rhythm, with significantly higher concentrations at night, reaching a median of 1.5 pg/mL (1.0–2.1) during the day compared to 7.3 pg/mL (3.8–13.6) at night, representing more than a five-fold increase. This rhythmicity may be important for the biological synchronization of the newborn and for protecting the gastrointestinal tract, particularly in preterm infants. Notably, in the milk of mothers of preterm infants, antioxidant enzymes also follow a circadian pattern: glutathione peroxidase 3 peaks at night, whereas SOD and total antioxidant capacity reach their highest levels during the day [78]. Melatonin present in colostrum modulates phagocyte function, even in the presence of *G. lamblia*. Its antioxidant and immunomodulatory effects have been observed in vitro, and higher melatonin concentrations were found in the colostrum of older mothers compared to younger women [79]. Although the exact source of melatonin in breast milk remains unclear, findings suggest that its presence may not be solely dependent on maternal blood levels. Significantly lower melatonin concentrations in milk compared to serum point toward the possibility of local synthesis within the mammary gland, although further studies are needed to confirm this hypothesis [78].

Patients infected with *G. lamblia* have more than twice the serum melatonin concentration compared to uninfected individuals [80]. Melatonin plays a key role in modulating the immune response during protozoan infections such as *G. lamblia*. In the study by de Queiroz et al. [81], women with recent *G. lamblia* infection (IgM+) had significantly lower melatonin concentrations in colostrum, whereas those with past infection (IgG+) exhibited higher levels. The authors suggest that melatonin levels in colostrum may reflect the phase of infection and influence the immunological environment, potentially affecting the course of giardiasis. In the study by Nasser et al. [82], melatonin encapsulated in lecithin/chitosan nanoparticles (Mel-LCNPs) resulted in complete elimination of *G. lamblia* cysts from the feces of infected mice by day 14. This formulation also normalized interleukin-4 and interferon-gamma levels to values comparable to those of healthy controls and demonstrated antioxidant and anti-inflammatory properties. These findings suggest that melatonin in nanoparticle form could represent a promising supportive approach for managing giardiasis, pending further clinical evaluation. Melatonin, as a neurohormone, may serve as an adjuvant therapy in the treatment of both intestinal and extraintestinal forms of amoebiasis. Under in vitro conditions, melatonin at 100 ng/mL (a pharmacological concentration commonly used to evaluate cellular activation) increased the adherence of *E. histolytica* trophozoites to leukocytes, including both polymorphonuclear leukocytes and mononuclear leukocytes, and also led to elevated levels of superoxide anion and SOD activity, indicating an enhancement of the phagocytes’ immune response [83].

## 8. Breast Milk as a Reservoir of Microorganisms That Positively Influence the Reduction in Protozoan Infections

Human milk is not a typical route of transmission for intestinal parasites such as *G. lamblia*, *E. histolytica*, or *Cryptosporidium* spp., nor for other tissue parasites such as *Toxoplasma gondii* and *Trypanosoma cruzi*, except in rare cases, for example, when blood is present in the milk during active infection in the mother [51,84]. Experimental studies in animals suggest that *Strongyloides stercoralis* may be transmitted via lactation, although this mechanism has not been confirmed in humans [84]. In veterinary literature, transmission of nematodes through milk is well documented, whereas in humans such cases remain inconclusive. In one mother, *Necator americanus* was detected in milk, and in 10% of newborns in southern Nigeria, nematode eggs were found in stool, despite the fact that no larvae were detected in the colostrum samples examined [51]. There are also indications that larvae may enter breast milk, as nematode eggs have been observed in the stools of infants too young to have had any direct contact with infective larvae [85]. The relationship between breastfeeding and parasite transmission, as well as its effect on infants, remains under investigation. More than a decade ago, Lawrence [51] emphasized that further epidemiological studies were needed to clarify the significance of parasite transmission through breast milk. To date, no substantial new evidence has emerged to change this perspective, and there are still no grounds for issuing specific recommendations regarding the safety of breastfeeding in the context of parasitic infections. According to the Centers for Disease Control and Prevention, absolute contraindications to breastfeeding remain restricted to selected viral infections (e.g., HIV, HTLV-1/2), active untreated tuberculosis, and certain bacterial infections such as untreated brucellosis, but do not include parasitic diseases [86].

The infant gut microbiome develops dynamically in the first months of life and is influenced by factors such as mode of delivery, type of feeding, use of antibiotics, and environmental conditions. Vaginal birth and breastfeeding promote colonization of the infant’s gut by bacteria of the genera *Bifidobacterium*, *Lactobacillus*, and *Streptococcus*, which dominate in early life and play a beneficial role in the development of the immune system. After the introduction of solid foods into the child’s diet, an increase in bacteria of the genera *Prevotella*, *Faecalibacterium*, and *Roseburia* is observed in the intestines, along with a decrease in the previously dominant bacteria [87]. *Prevotella copri* (recently reclassified as *Segatella copri*) is the predominant *Prevotella* species within the human gut microbiome, [88,89]. In infants, it is identified as the dominant taxon in about half of samples, where it represents more than 80% of the *Prevotella* genus abundance [89]. This species is believed to play a role in the metabolism of plant fiber in humans. Importantly, studies conducted among children from low-income urban environments have shown that an increased abundance of this bacterium was significantly associated with diarrhea caused by *E. histolytica* in the second year of life, suggesting that its dominance in the gut microbiota may influence the symptomatic course of infection [14]. Infections caused by *G. intestinalis* correlate with the presence of dysbiosis, manifested by increased abundance of potentially harmful bacteria such as *Escherichia coli* and a disturbed balance of the microbiota [15]. Studies in animal models have also shown that *G. lamblia* infection leads to profound changes in the composition of the gut microbiota and disturbances in bile acid metabolism, resulting in improper absorption of nutrients, disrupted metabolism, and impaired growth [16]. Giardiasis may lead to a decrease in bacteria considered protective, such as *Bifidobacterium* and *Lactobacillus* [90]. It can therefore be assumed that breastfeeding, by supporting the predominance of protective *Bifidobacterium* spp. and *Lactobacillus* spp., plays a role in reducing the risk of symptomatic protozoan infections.

## 9. Maternal Diet and the Bioactivity of Human Milk

Emerging evidence supports that maternal consumption of fermentable fibers and prebiotics—such as inulin and fructooligosaccharides—modulates the maternal gut microbiota, enhancing mucin production in the gastrointestinal tract and subsequently influencing the immunological composition of breast milk. Maternal fructooligosaccharides supplementation has been demonstrated to induce compositional shifts in the human milk microbiota, although individual responses may vary. A cohort analysis found that higher dietary fiber intake in lactating women correlated with increased microbial diversity and favorable shifts in milk-associated bacterial communities, suggesting a potential influence on mucin levels and immunoglobulin concentrations in milk [91]. Furthermore, a recent review emphasized that modulation of maternal gut health through diet or probiotic intake may indirectly affect the composition of breast milk, including HMOs and secretory IgA [91,92]. Fermentation of dietary fiber by the gut microbiota, particularly by genera such as *Bifidobacterium* and *Lactobacillus*, leads to the production of short-chain fatty acids (SCFA), namely acetate, propionate, and butyrate. These metabolites upregulate the expression of mucin-encoding genes, particularly MUC2, through several well-characterized mechanisms. Butyrate functions as a histone deacetylase (HDAC) inhibitor, enhancing histone acetylation at the MUC2 promoter and thereby stimulating its transcription. In addition, butyrate activates the epidermal growth factor receptor (EGFR)/ERK MAPK signaling pathway in goblet cells, further promoting MUC2 expression. Moreover, SCFA signaling through GPR43 and GPR109A contributes to mucin secretion and strengthening of the intestinal mucus barrier [93,94,95,96,97]. While butyrate can modulate NF-κB activity, studies show that it typically inhibits NF-κB and histone deacetylation, and NF-κB is not the primary pathway driving MUC2 induction; instead, HDAC inhibition and EGFR/ERK MAPK activation are the dominant mechanisms [98,99].These local effects on gut mucosa extend systemically, as SCFAs and immune mediators can enter the circulation and potentially influence the mammary gland microenvironment, thereby modifying the profile of bioactive molecules secreted into breast milk, including mucin-associated glycoproteins. Although direct human studies measuring mucin levels in breast milk post maternal fiber intake are limited, recent research establishes that higher maternal dietary fiber correlates with increased diversity of milk microbiota and favorable functional profiles [94].

In addition, maternal consumption of fermented foods—such as yogurt, kefir, sauerkraut, and other traditionally cultured products has been shown to beneficially modulate the maternal gut microbiota, thereby indirectly influencing the composition of HMOs and the antibody repertoire in breast milk. Emerging research indicates that specific bacterial strains, particularly from the *Lactobacillus* spp. and *Bifidobacterium* spp., are associated with favorable changes in milk composition, including increased levels of sialylated and fucosylated HMOs and elevated concentrations of sIgA [100,101,102]. These fermented foods serve as sources of probiotics and postbiotics that promote the proliferation of beneficial microbes in the maternal gut. Intervention studies have demonstrated that diets rich in fermented products enhance maternal microbial diversity and reduce markers of systemic inflammation, thereby supporting intestinal barrier function and the transfer of bioactive compounds into breast milk [101,103,104]. Specifically, consumption of fermented foods has been shown to significantly increase alpha diversity indices; in a 10-week dietary intervention, participants consuming a diet rich in fermented foods exhibited a measurable rise in the Shannon index and Faith’s phylogenetic diversity compared to baseline, indicating an expansion of microbial richness and evenness [105]. Although the precise mechanisms through which microbial and dietary factors influence the mammary gland are still under investigation, it is hypothesized that gut-derived metabolites—such as SCFAs and bioactive peptides—may reach the mammary tissue via systemic circulation and modulate the breast milk microenvironment [106]. These findings support the growing notion that diet-driven improvement of maternal gut health, particularly through the regular intake of fermented foods, may beneficially impact neonatal immune development and protection against enteric pathogens.

To summarize, a breastfeeding woman should consume fermented foods daily, such as natural yogurt, kefir, and fermented vegetables (e.g., sauerkraut, pickles), as they provide probiotics that support a healthy gut microbiota. Simultaneously, a diet rich in fermentable fibers and prebiotics—found in onions, garlic, leeks, chicory, fruits, legumes, and whole grains, is recommended to promote the growth of beneficial bacteria and the production of SCFAs. It is also advisable to avoid highly processed foods. For optimal immune support and breast milk quality, proper hydration, regular intake of fatty fish (e.g., salmon, mackerel), and a fresh, varied diet are essential.

## 10. Maternal Nutritional Status and Immunoprotective Quality of Breast Milk

Maternal nutritional status plays a pivotal role in shaping the immunological composition of breast milk. Protein-energy malnutrition (PEM) and deficiencies in critical micronutrients, such as iron, zinc, and vitamin A can adversely affect concentrations of lactoferrin, sIgA, and HMOs, all of which are key in defending infants against enteric protozoal pathogens [107,108]. Studies in undernourished women have demonstrated that those experiencing PEM, vitamin A deficiency, or iron-deficiency anemia produce breast milk with significantly lower sIgA levels compared to well-nourished counterparts [107]. Similarly, protein-energy malnutrition has been linked to reduced mammary lactoferrin synthesis [107]. Although direct evidence on how micronutrient deficiencies influence HMO profiles is limited, existing data underscore the importance of adequate maternal nutrition for maintaining optimal milk quality [109]. Given the protective roles of these bioactives against pathogens like *G. lamblia* and *E. histolytica*, it is critical to highlight maternal nutritional status as a modifiable determinant of milk-mediated infant immunity [110]. Table 2 summarizes the effects of specific maternal nutrient deficiencies on immune components in human milk and their associated immunological consequences.

## 11. Lifestyle Related Modulation of Milk Composition

Maternal lifestyle factors, particularly psychological stress, sleep quality, and physical activity—are increasingly recognized as important modulators of the bioactive composition of human milk. Melatonin, a hormone with immunomodulatory, antioxidant, and circadian-regulating properties, exhibits a clear diurnal rhythm in breast milk that mirrors the mother’s sleep–wake cycle. Disrupted or insufficient maternal sleep, especially during the postpartum period, may lead to reduced melatonin secretion, potentially weakening its protective effects on the infant’s immune function and gut barrier integrity [120,121]. Psychological stress, both acute and chronic, is associated with elevated cortisol and systemic proinflammatory cytokines, which can alter the immune profile of breast milk. Maternal psychological stress has been associated with alterations in breast milk immune composition. Several studies report that higher perceived stress in mothers correlates with reduced concentrations of secretory IgA (sIgA) in milk, suggesting a stress-related impairment of mucosal immune transfer to the infant [122,123,124]. However, while the direction of this association is consistent, the literature does not provide reliable quantitative estimates of percentage reduction in sIgA levels between high-stress and low-stress mothers. Such shifts may impair the milk’s mucosal protective functions, especially against gastrointestinal pathogens such as *G. lamblia* and *E. histolytica*. In contrast, moderate physical activity exerts anti-inflammatory and immune-supportive effects. In lactating women, regular exercise has been linked to improved cytokine profiles, enhanced lymphocyte function, and increased levels of protective antibodies, including IgA. Although direct data remain limited, emerging evidence suggests that maternal physical activity may positively influence the immunological quality of milk [125]. Moderate physical activity during lactation appears safe and does not impair maternal immune status [126]. Although direct data on immune-enhancing effects in lactating women are limited, studies in general female populations show that acute bouts of exercise increase circulating CD8^+^ T lymphocytes and CD56^+^CD16^+^ natural killer (NK) cells in peripheral blood [127,128], suggesting potential immune benefits for breastfeeding mothers.

These findings underscore the importance of a holistic approach to maternal well-being during lactation. Promoting adequate sleep, stress reduction, and healthy physical activity not only benefits maternal health but may also enhance the immunoprotective potential of breast milk, which is an especially relevant factor in preventing parasitic infections in early infancy.

## 12. Suggestions for Future Research

Given the multifactorial nature of human milk bioactivity and the complexity of host–parasite interactions in early infancy, further research is warranted to elucidate how maternal nutrition influences protective factors in breast milk and infant health outcomes. Several areas emerge as particularly promising:

1. Observational cohort studies investigating the relationship between maternal dietary patterns, particularly fiber intake, probiotic consumption, and overall nutrient density—and the incidence of intestinal protozoal infections (e.g., *G. lamblia*, *E. histolytica*) in exclusively breastfed infants. Such studies could help clarify whether maternal diet directly modulates infant susceptibility to parasitic infections through changes in the milk’s immunological profile.
2. Controlled dietary intervention trials assessing the effects of specific maternal nutritional interventions (e.g., high-fiber diets, probiotic supplementation, or vitamin D intake) on the concentration and activity of mucosal defense-related components in milk, such as mucins (e.g., MUC1, MUC2) and lactoferrin. Longitudinal measurements of these bioactives in breast milk, combined with clinical follow-up of infant health outcomes, would help establish causal links between diet and milk-mediated immunoprotection.
3. Mechanistic studies exploring how maternal gut-derived metabolites, such as SCFAs or bioactive peptides, are transferred to the mammary gland and influence the synthesis or secretion of immune factors in milk.

Such research would not only deepen our understanding of maternal–infant nutritional immunology but could also inform evidence-based dietary recommendations for lactating women aimed at maximizing the anti-parasitic and mucosal-protective properties of human milk.

## 13. Conclusions

**Figure 1:**
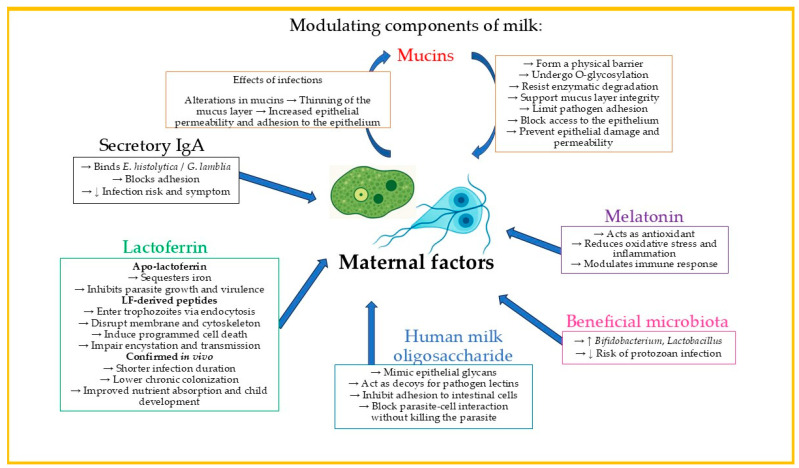
Maternal milk factors contributing to protection against intestinal protozoan infections in infants.

Human milk is a biologically complex fluid that provides essential immunological protection to infants, particularly against intestinal protozoan infections such as *G. lamblia* and *E. histolytica*. Its bioactive components, including mucins, lactoferrin, human milk oligosaccharides, melatonin, and sIgA, contribute to the maintenance of intestinal barrier function, the modulation of immune responses, and the limitation of pathogen adhesion. Maternal nutritional status and lifestyle factors can influence the composition and functionality of these components, potentially affecting the protective capacity of breast milk. Promoting maternal well-being and exclusive breastfeeding may therefore be crucial in reducing the burden of protozoan infections in infancy. Figure 1 below summarizes the main maternal milk-derived factors involved in protecting infants against intestinal protozoan infections through various antimicrobial, immunomodulatory, and barrier-stabilizing mechanisms.

## Figures and Tables

**Table 1:** Protective efficacy of breast milk antibodies against diarrheal and asymptomatic protozoal infections in infants.

Parasite	Type of Evidence	Presence and Specificity of Antibodies	Observed Effect	Ref.
*Entamoeba histolytica*	Mother–infant observational study	sIgA in milk and saliva	Despite 67% of mothers being infected, only 2 infant samples were positive; suggesting potential protection via breastfeeding (no statistical analysis was reported)	[59]
*Entamoeba histolytica*	Direct analysis of breast milk	CRD-specific sIgA (Gal/GalNAc lectin)	Higher breast milk sIgA levels were associated with reduced risk of infection and 64% lower risk of amebic diarrhea (HR = 0.356; 95% CI: 0.149–0.849; *p* = 0.020)	[60]
*Giardia lamblia*	Direct analysis of breast milk	sIgA specific to recombinant cyst wall protein	No association between breast milk sIgA levels and infection or diarrhea (no statistical analysis was reported)	[60]
*Entamoeba histolytica*	Comparison: breast-fed vs. non-breast-fed	Serum IgE	Lower infection rate (*p* < 0.05) and lower IgE (*p* < 0.01) in infected breast-fed infants compared to infected non-breast-fed	[21]
*Giardia lamblia*	Comparison: breast-fed vs. non-breast-fed	Serum IgE	Lower infection rate (*p* < 0.05) and lower IgE (*p* < 0.01) in infected breast-fed infants compared to infected non-breast-fed)	[21]

Carbohydrate recognition domain (CRD).

**Table 2:** Effects of maternal nutrient deficiencies on immune components in human milk [107,108].

Nutrient Deficiency	Presence and Specificity of Antibodies	Immunological/Clinical Consequence
Protein-energy malnutrition	↓ LF, ↓ sIgA	Reduced antimicrobial and gut-protective activity; associated with increased risk and severity of diarrheal illness/persistence [111,112]
Zinc deficiency	↓ mucin synthesis, ↓ sIgA	Weakened mucosal barrier; increased susceptibility to giardiasis and diarrheal disease; zinc supplementation can reduce *Giardia*-associated diarrhea in some settings [113,114,115]
Vitamin A deficiency	↓ sIgA, compromised mucosal immunity	Altered epithelial integrity and immune regulation; higher diarrheal morbidity reported in several cohorts; VAS reduces diarrhea incidence in meta-analyses (heterogeneous effects across studies) [116,117]
Iron deficiency	↓ LF functionality	Lowered pathogen iron sequestration and impaired mucosal defenses; giardiasis is associated with iron deficiency; quantitative risk increase for giardiasis in iron-deficient infants is not well established [118,119]

Lactoferrin (LF); Vitamin A supplementation (VAS).

## Abbreviations

The following abbreviations are used in this manuscript:

CD	Cluster of differentiation
CRD	Carbohydrate recognition domain
EGFR/ERK MAPK	Epidermal growth factor receptor/extracellular signal-regulated kinase mitogen-activated protein kinase
Gal	Galactose
GalNAc	N-acetylgalactosamine
GPR	G protein-coupled receptor
HDAC	Histone deacetylase
HMO	Human milk oligosaccharides
IgA	Immunoglobulin A
IL-6	Interleukin 6
LFampin	Lactoferrampin
LFcin	Lactoferricin
LF	Lactoferrin
Mel-LCNPs	Melatonin-loaded nanoparticles composed of lecithin and chitosan
MUC	Mucin
NF- κB	Nuclear factor kappa B
NK	Natural killer cells
PEM	Protein-energy malnutrition
ROS	Reactive oxygen species
sIgA	Secretory immunoglobulin A
SCFA	Short-chain fatty acids
SOD	Superoxide dismutase
TNF-α	Tumor necrosis factor alpha
VAS	Vitamin A supplementation
VSPs	Variant-specific surface protein
ZO-1	Zonula occludens-1

## References

[1] Meng F., Uniacke-Lowe T., Ryan A.C., Kelly A.L. (2021). The Composition and Physico-Chemical Properties of Human Milk: A Review. Trends Food Sci. Technol..

[2] Turin C.G., Ochoa T.J. (2014). The Role of Maternal Breast Milk in Preventing Infantile Diarrhea in the Developing World. Curr. Trop. Med. Rep..

[3] Özdemir S.A., Şahin Ö.N., Briana D.D. (2023). Human Milk Composition: Nutrients and Bioactive Factors. Breastfeeding and Metabolic Programming.

[4] Berkhout M.D., Ioannou A., Kavanal Jayaprakash Y., Plugge C.M., Belzer C. (2025). Milk and Mucin Glycans Orchestrate a Synthetic Infant Gut Microbiota Structure. FEMS Microbiol. Ecol..

[5] France T.C., Kennedy E., O’Regan J., Goulding D.A. (2024). Current Perspectives on the Use of Milk Fat Globule Membrane in Infant Milk Formula. Crit. Rev. Food Sci. Nutr..

[6] Peterson J.A., Hamosh M., Scallan C.D., Ceriani R.L., Henderson T.R., Mehta N.R., Armand M., Hamosh P. (1998). Milk Fat Globule Glycoproteins in Human Milk and in Gastric Aspirates of Mother’s Milk-Fed Preterm Infants. Pediatr. Res..

[7] Solaymani-Mohammadi S. (2022). Mucosal Defense Against Giardia at the Intestinal Epithelial Cell Interface. Front. Immunol..

[8] Bode L., Jantscher-Krenn E. (2012). Structure-Function Relationships of Human Milk Oligosaccharides. Adv. Nutr..

[9] Bührer C., Ensenauer R., Jochum F., Kalhoff H., Koletzko B., Lawrenz B., Mihatsch W., Posovszky C., Rudloff S. (2022). Infant Formulas with Synthetic Oligosaccharides and Respective Marketing Practices: Position Statement of the German Society for Child and Adolescent Medicine e.V. (DGKJ), Commission for Nutrition. Mol. Cell. Pediatr..

[10] Szyller H., Antosz K., Batko J., Mytych A., Dziedziak M., Wrześniewska M., Braksator J., Pytrus T. (2024). Bioactive Components of Human Milk and Their Impact on Child’s Health and Development, Literature Review. Nutrients.

[11] Le Doare K., Holder B., Bassett A., Pannaraj P.S. (2018). Mother’s Milk: A Purposeful Contribution to the Development of the Infant Microbiota and Immunity. Front. Immunol..

[12] Dombrowska-Pali A., Wiktorczyk-Kapischke N., Chrustek A., Olszewska-Słonina D., Gospodarek-Komkowska E., Socha M.W. (2024). Human Milk Microbiome—A Review of Scientific Reports. Nutrients.

[13] Boix-Amorós A., Collado M.C., Mira A. (2016). Relationship between Milk Microbiota, Bacterial Load, Macronutrients, and Human Cells during Lactation. Front. Microbiol..

[14] Gilchrist C.A., Petri S.E., Schneider B.N., Reichman D.J., Jiang N., Begum S., Watanabe K., Jansen C.S., Elliott K.P., Burgess S.L. (2016). Role of the Gut Microbiota of Children in Diarrhea Due to the Protozoan Parasite *Entamoeba histolytica*. J. Infect. Dis..

[15] Iebba V., Santangelo F., Totino V., Pantanella F., Monsia A., Di Cristanziano V., Di Cave D., Schippa S., Berrilli F., D’Alfonso R. (2016). Gut Microbiota Related to *Giardia duodenalis*, *Entamoeba* spp. and *Blastocystis hominis* Infections in Humans from Côte d’Ivoire. J. Infect. Dev. Ctries..

[16] Riba A., Hassani K., Walker A., van Best N., von Zeschwitz D., Anslinger T., Sillner N., Rosenhain S., Eibach D., Maiga-Ascofaré O. (2020). Disturbed Gut Microbiota and Bile Homeostasis in Giardia -Infected Mice Contributes to Metabolic Dysregulation and Growth Impairment. Sci. Transl. Med..

[17] Ahmed M. (2023). Intestinal Parasitic Infections in 2023. Gastroenterol. Res..

[18] Weström B., Arévalo Sureda E., Pierzynowska K., Pierzynowski S.G., Pérez-Cano F.-J. (2020). The Immature Gut Barrier and Its Importance in Establishing Immunity in Newborn Mammals. Front. Immunol..

[19] Collado M.C., Cernada M., Neu J., Pérez-Martínez G., Gormaz M., Vento M. (2015). Factors Influencing Gastrointestinal Tract and Microbiota Immune Interaction in Preterm Infants. Pediatr. Res..

[20] Klotz C., Aebischer T. (2015). The Immunological Enigma of Human Giardiasis. Curr. Trop. Med. Rep..

[21] Abdel-Hafeez E.H., Belal U.S., Abdellatif M.Z.M., Naoi K., Norose K. (2013). Breast-Feeding Protects Infantile Diarrhea Caused by Intestinal Protozoan Infections. Korean J. Parasitol..

[22] Sardinha-Silva A., Alves-Ferreira E.V.C., Grigg M.E. (2022). Intestinal Immune Responses to Commensal and Pathogenic Protozoa. Front. Immunol..

[23] Astiazaran-Garcia H., Lopez-Teros V., Valencia M.E., Vazquez-Ortiz F., Sotelo-Cruz N., Quihui-Cota L. (2010). *Giardia lamblia* Infection and Its Implications for Vitamin A Liver Stores in School Children. Ann. Nutr. Metab..

[24] Takahashi M., Katayama Y., Takada H., Hirakawa J., Kuwayama H., Yamaji H., Ogura K., Meda S., Omata M. (2001). Silent Infection of *Giardia lamblia* Causing Bleeding through Vitamin K Malabsorption. J. Gastroenterol. Hepatol..

[25] Cordingley F.T., Crawford G.P.M. (1986). Giardia Infection Causes Vitamin B12 Deficiency. Aust. N. Z. J. Med..

[26] Sandoval-Ramírez T., Seco-Hidalgo V., Calderon-Espinosa E., Garcia-Ramon D., Lopez A., Calvopiña M., Guadalupe I., Chico M., Mejia R., Chis Ster I. (2023). Epidemiology of Giardiasis and Assemblages A and B and Effects on Diarrhea and Growth Trajectories during the First 8 Years of Life: Analysis of a Birth Cohort in a Rural District in Tropical Ecuador. PLoS Negl. Trop. Dis..

[27] Tellez A., Winiecka-Krusnell J., Paniagua M., Linder E. (2003). Antibodies in Mother’s Milk Protect Children against Giardiasis. Scand. J. Infect. Dis..

[28] Dhubyan Mohammed Zaki Z. (2022). Prevalence of *Entamoeba histolytica* and *Giardia lamblia* Associated with Diarrhea in Children Referring to Lbn Al-Atheer Hospital in Mosul, Iraq. Arch. Razi Inst..

[29] Aguiar A., Saraiva S., Pontes M., Coelho E. (2011). Eosinophilia in a Newborn: A Case of Giardiasis and Milk Allergy. Acta Med. Port..

[30] Adam R.D. (2021). *Giardia duodenalis*: Biology and Pathogenesis. Clin. Microbiol. Rev..

[31] Liu J., Ma’ayeh S., Peirasmaki D., Lundström-Stadelmann B., Hellman L., Svärd S.G. (2018). Secreted *Giardia intestinalis* Cysteine Proteases Disrupt Intestinal Epithelial Cell Junctional Complexes and Degrade Chemokines. Virulence.

[32] Fekete E., Allain T., Sosnowski O., Anderson S., Lewis I.A., Buret A.G. (2024). *Giardia* spp.-Induced Microbiota Dysbiosis Disrupts Intestinal Mucin Glycosylation. Gut Microbes.

[33] Zibaei M., Firoozeh F., Azargoon A. (2012). Infantile Amoebiasis: A Case Report. Case Rep. Infect. Dis..

[34] Güven A. (2003). Amebiasis in the Newborn. Indian J. Pediatr..

[35] Uddin M.J., Leslie J.L., Petri W.A. (2021). Host Protective Mechanisms to Intestinal Amebiasis. Trends Parasitol..

[36] Mondal D., Haque R., Sack R.B., Kirkpatrick B.D., Petri W.A. (2009). Attribution of Malnutrition to Cause-Specific Diarrheal Illness: Evidence from a Prospective Study of Preschool Children in Mirpur, Dhaka, Bangladesh. Am. J. Trop. Med. Hyg..

[37] Magon P. (2010). Neonatal Amoebiasis. Indian J. Pediatr..

[38] Espinosa-Cantellano M., Martínez-Palomo A. (2000). Pathogenesis of Intestinal Amebiasis: From Molecules to Disease. Clin. Microbiol. Rev..

[39] Kahng J., Kim S.-Y. (2007). A Case of Neonatal Amoebiasis with After-Birth Vomiting and Bloody Stool. Korean J. Pediatr..

[40] Jantscher-Krenn E., Lauwaet T., Bliss L.A., Reed S.L., Gillin F.D., Bode L. (2012). Human Milk Oligosaccharides Reduce *Entamoeba histolytica* Attachment and Cytotoxicity in Vitro. Br. J. Nutr..

[41] Labruyère E., Thibeaux R., Olivo-Marin J.-C., Guillén N. (2019). Crosstalk between *Entamoeba histolytica* and the Human Intestinal Tract during Amoebiasis. Parasitology.

[42] Betanzos A., Javier-Reyna R., García-Rivera G., Bañuelos C., González-Mariscal L., Schnoor M., Orozco E. (2013). The EhCPADH112 Complex of *Entamoeba histolytica* Interacts with Tight Junction Proteins Occludin and Claudin-1 to Produce Epithelial Damage. PLoS ONE.

[43] Hansson G.C. (2020). Mucins and the Microbiome. Annu. Rev. Biochem..

[44] Roskens H., Erlandsen S.L. (2002). Inhibition of in Vitro Attachment of *Giardia trophozoites* by Mucin. J. Parasitol..

[45] Zenian A., Gillin F.D. (1985). Interactions of *Giardia lamblia* with Human Intestinal Mucus: Enhancement of Trophozoite Attachment to Glass 1. J. Protozool..

[46] Zenian A.J., Gillin F. (1987). Intestinal Mucus Protects *Giardia lamblia* from Killing by Human Milk. J. Protozool..

[47] Amat C.B., Motta J.P., Fekete E., Moreau F., Chadee K., Buret A.G. (2017). Cysteine Protease–Dependent Mucous Disruptions and Differential Mucin Gene Expression in *Giardia duodenalis* Infection. Am. J. Pathol..

[48] Hicks S.J., Theodoropoulos G., Carrington S.D., Corfield A.P. (2000). The Role of Mucins in Host-Parasite Interactions: Part I—Protozoan Parasites. Parasitol. Today.

[49] Cuellar P., Hernández-Nava E., García-Rivera G., Chávez-Munguía B., Schnoor M., Betanzos A., Orozco E. (2017). *Entamoeba histolytica* EhCP112 Dislocates and Degrades Claudin-1 and Claudin-2 at Tight Junctions of the Intestinal Epithelium. Front. Cell. Infect. Microbiol..

[50] Dey I., Chadee K. (2008). Prostaglandin E 2 Produced by *Entamoeba histolytica* Binds to EP4 Receptors and Stimulates Interleukin-8 Production in Human Colonic Cells. Infect. Immun..

[51] Lawrence R.M. (2011). Transmission of Infectious Diseases Through Breast Milk and Breastfeeding. Breastfeeding.

[52] Mahmud M.A., Chappell C.L., Hossain M.M., Huang D.B., Habib M., DuPont H.L. (2001). Impact of Breast-Feeding on *Giardia lamblia* Infections in Bilbeis, Egypt. Am. J. Trop. Med. Hyg..

[53] Morrow A.L., Reves R.R., West M.S., Guerrero M.L., Ruiz-Palacios G.M., Pickering L.K. (1992). Protection against Infection with *Giardia lamblia* by Breast-Feeding in a Cohort of Mexican Infants. J. Pediatr..

[54] Akisu C. (2004). Effect of Human Milk and Colostrum on *Entamoeba histolytica*. World J. Gastroenterol..

[55] Haque R., Mondal D., Duggal P., Kabir M., Roy S., Farr B.M., Sack R.B., Petri W.A. (2006). *Entamoeba histolytica* Infection in Children and Protection from Subsequent Amebiasis. Infect. Immun..

[56] Guerrero-Manríquez G.G., Sánchez-Ibarra F., Avila E.E. (1998). Inhibition of *Entamoeba histolytica* Proteolytic Activity by Human Salivary IgA Antibodies. APMIS.

[57] Leon-Sicairos N., Lopez-Soto F., Reyes-Lopez M., Godinez-Vargas D., Ordaz-Pichardo C., de la Garza M. (2006). Amoebicidal Activity of Milk, Apo-Lactoferrin, SIgA and Lysozyme. Clin. Med. Res..

[58] Téllez A., Palm D., Weiland M., Alemán J., Winiecka-Krusnell J., Linder E., Svärd S. (2005). Secretory Antibodies against *Giardia intestinalis* in Lactating Nicaraguan Women. Parasite Immunol..

[59] Islam A., Stoll B.J., Ljungstrom I., Biswas J., Nazrul H., Huldt G. (1988). The Prevalence of *Entamoeba histolytica* in Lactating Women and in Their Infants in Bangladesh. Trans. R. Soc. Trop. Med. Hyg..

[60] Korpe P.S., Liu Y., Siddique A., Kabir M., Ralston K., Ma J.Z., Haque R., Petri W.A. (2013). Breast Milk Parasite-Specific Antibodies and Protection From Amebiasis and Cryptosporidiosis in Bangladeshi Infants: A Prospective Cohort Study. Clin. Infect. Dis..

[61] Newburg D.S., Ruiz-Palacios G.M., Altaye M., Chaturvedi P., Meinzen-Derr J., de Lourdes Guerrero M., Morrow A.L. (2004). Innate Protection Conferred by Fucosylated Oligosaccharides of Human Milk against Diarrhea in Breastfed Infants. Glycobiology.

[62] Newburg D.S. (2013). Glycobiology of Human Milk. Biochemistry.

[63] Smilowitz J.T., Lebrilla C.B., Mills D.A., German J.B., Freeman S.L. (2014). Breast Milk Oligosaccharides: Structure-Function Relationships in the Neonate. Annu. Rev. Nutr..

[64] Cano-Mancera R., López-Revilla R. (1987). Inhibition of the Adhesion of *Entamoeba histolytica* Trophozoites to Human Erythrocytes by Carbohydrates. Parasitol. Res..

[65] Al-Azzam S. (2018). The Effect of Human Milk Oligosaccharides on Neglected Infectious Diseases. Master’s Thesis.

[66] Długosz A., Wróblewska J., Kołaczyk P., Wróblewska W. (2025). The Role of Lactoferrin in Combating *Candida* spp. Infections Through Regulation of Oxidative Stress, Immune Response, and Nutritional Support in Women and Newborns. Molecules.

[67] Villavicencio A., Rueda M.S., Turin C.G., Ochoa T.J. (2017). Factors Affecting Lactoferrin Concentration in Human Milk: How Much Do We Know?. Biochem. Cell Biol..

[68] Czosnykowska-Łukacka M., Orczyk-Pawiłowicz M., Broers B., Królak-Olejnik B. (2019). Lactoferrin in Human Milk of Prolonged Lactation. Nutrients.

[69] Frontera L.S., Moyano S., Quassollo G., Lanfredi-Rangel A., Rópolo A.S., Touz M.C. (2018). Lactoferrin and Lactoferricin Endocytosis Halt Giardia Cell Growth and Prevent Infective Cyst Production. Sci. Rep..

[70] Anand N. (2024). Antiparasitic Activity of the Iron-Containing Milk Protein Lactoferrin and Its Potential Derivatives against Human Intestinal and Blood Parasites. Front. Parasitol..

[71] Turchany J.M., Mccaffery J.M., Aley S.B., Gillin F.D. (1997). Ultrastructural Effects of Lactoferring Binding on *Giardia lamblia* Trophozoites. J. Eukaryot. Microbiol..

[72] Aguilar-Diaz H., Canizalez-Roman A., Nepomuceno-Mejia T., Gallardo-Vera F., Hornelas-Orozco Y., Nazmi K., Bolscher J.G.M., Carrero J.C., Leon-Sicairos C., Leon-Sicairos N. (2017). Parasiticidal Effect of Synthetic Bovine Lactoferrin Peptides on the Enteric Parasite *Giardia intestinalis*. Biochem. Cell Biol..

[73] Ochoa T.J., Chea-Woo E., Campos M., Pecho I., Prada A., McMahon R.J., Cleary T.G. (2008). Impact of Lactoferrin Supplementation on Growth and Prevalence of Giardia Colonization in Children. Clin. Infect. Dis..

[74] Ordaz-Pichardo C., León-Sicairos N., Hernández-Ramírez V.I., Talamás-Rohana P., de la Garza M. (2012). Effect of Bovine Lactoferrin in a Therapeutic Hamster Model of Hepatic Amoebiasis 1 This Article Is Part of a Special Issue Entitled Lactoferrin and Has Undergone the Journal’s Usual Peer Review Process. Biochem. Cell Biol..

[75] Reyes-López M., Ramírez-Rico G., Serrano-Luna J., de la Garza M. (2022). Activity of Apo-Lactoferrin on Pathogenic Protozoa. Pharmaceutics.

[76] Kopustinskiene D.M., Bernatoniene J. (2021). Molecular Mechanisms of Melatonin-Mediated Cell Protection and Signaling in Health and Disease. Pharmaceutics.

[77] Chrustek A., Sinkiewicz-Darol E., Lampka M., Olszewska-Słonina D., Sperkowska B., Linowiecka K. (2022). Effect of Pasteurization on Melatonin Concentration in Human Breast Milk. Postep. Hig. Med. Dosw..

[78] Katzer D., Pauli L., Mueller A., Reutter H., Reinsberg J., Fimmers R., Bartmann P., Bagci S. (2016). Melatonin Concentrations and Antioxidative Capacity of Human Breast Milk According to Gestational Age and the Time of Day. J. Hum. Lact..

[79] Pereira Q.L.C., de Castro Pernet Hara C., Fernandes R.T.S., Fagundes D.L.G., do Carmo França-Botelho A., Gomes M.A., França E.L., Honorio-França A.C. (2018). Human Colostrum Action against *Giardia lamblia* Infection Influenced by Hormones and Advanced Maternal Age. Parasitol. Res..

[80] Al-Hadraawy S.K., Al-ghurabi M.E., Al-musawi M.M., Alzeyadi M. (2016). Ghrelin and Melatonin as Biomarkers in Patients with Giardiasis. Biotechnol. Biotechnol. Equip..

[81] de Queiroz A.A., França E.L., Gadenz G.R.B., Dalcin L.D.L., Fujimori M., França D.C.H., Gomes M.A., Honorio-França A.C. (2024). Correlation between Melatonin and Colostral Regulatory T Cells in *Giardia lamblia* Infection. Biomolecules.

[82] Nasser M., El-atif M.B.A., Alaa H., Abdelaziz M., Mustafa M., Masour M., Magdy S., Mohsen S., El Karamany Y., Farid A. (2025). Discovering the Anti-Parasitic Activity of Melatonin Loaded Lecithin/Chitosan Nanoparticles against Giardiasis and Cryptosporidiosis in Balb/c Infected Mice. Beni-Suef Univ. J. Basic Appl. Sci..

[83] França-Botelho A.C., França J.L., Oliveira F.M.S., Franca E.L., Honório-França A.C., Caliari M.V., Gomes M.A. (2011). Melatonin Reduces the Severity of Experimental Amoebiasis. Parasit. Vectors.

[84] Kutty P.K. (2014). Breastfeeding and Risk of Parasitic Infection-a Review. Asian Pac. J. Trop. Biomed..

[85] Hall A., Hewitt G., Tuffrey V., De Silva N. (2008). A Review and Meta-analysis of the Impact of Intestinal Worms on Child Growth and Nutrition. Matern. Child Nutr..

[86] Contraindications to Breastfeeding. https://www.cdc.gov/breastfeeding-special-circumstances/hcp/contraindications/index.html?utm_source=chatgpt.com.

[87] Bhattacharyya C., Barman D., Tripathi D., Dutta S., Bhattacharya C., Alam M., Choudhury P., Devi U., Mahanta J., Rasaily R. (2023). Influence of Maternal Breast Milk and Vaginal Microbiome on Neonatal Gut Microbiome: A Longitudinal Study during the First Year. Microbiol. Spectr..

[88] Gao Y., Nanan R., Macia L., Tan J., Sominsky L., Quinn T.P., O’Hely M., Ponsonby A.-L., Tang M.L.K., Collier F. (2021). The Maternal Gut Microbiome during Pregnancy and Offspring Allergy and Asthma. J. Allergy Clin. Immunol..

[89] Han N., Peng X., Zhang T., Qiang Y., Li X., Zhang W. (2024). Temporal Dynamics and Species-Level Complexity of *Prevotella* spp. in the Human Gut Microbiota: Implications for Enterotypes and Health. Front. Microbiol..

[90] Fekete E., Allain T., Siddiq A., Sosnowski O., Buret A.G. (2021). *Giardia* spp. and the Gut Microbiota: Dangerous Liaisons. Front. Microbiol..

[91] Sun W., Tao L., Qian C., Xue P., Du S., Tao Y. (2025). Human Milk Oligosaccharides: Bridging the Gap in Intestinal Microbiota between Mothers and Infants. Front. Cell. Infect. Microbiol..

[92] Desai M.S., Seekatz A.M., Koropatkin N.M., Kamada N., Hickey C.A., Wolter M., Pudlo N.A., Kitamoto S., Terrapon N., Muller A. (2016). A Dietary Fiber-Deprived Gut Microbiota Degrades the Colonic Mucus Barrier and Enhances Pathogen Susceptibility. Cell.

[93] Overby H.B., Ferguson J.F. (2021). Gut Microbiota-Derived Short-Chain Fatty Acids Facilitate Microbiota:Host Cross Talk and Modulate Obesity and Hypertension. Curr. Hypertens. Rep..

[94] Nogal A., Valdes A.M., Menni C. (2021). The Role of Short-Chain Fatty Acids in the Interplay between Gut Microbiota and Diet in Cardio-Metabolic Health. Gut Microbes.

[95] Berni Canani R., Di Costanzo M., Leone L. (2012). The Epigenetic Effects of Butyrate: Potential Therapeutic Implications for Clinical Practice. Clin. Epigenetics.

[96] Docampo M.D., da Silva M.B., Lazrak A., Nichols K.B., Lieberman S.R., Slingerland A.E., Armijo G.K., Shono Y., Nguyen C., Monette S. (2022). Alloreactive T Cells Deficient of the Short-Chain Fatty Acid Receptor GPR109A Induce Less Graft-versus-Host Disease. Blood.

[97] Jung T.-H., Park J.H., Jeon W.-M., Han K.-S. (2015). Butyrate Modulates Bacterial Adherence on LS174T Human Colorectal Cells by Stimulating Mucin Secretion and MAPK Signaling Pathway. Nutr. Res. Pract..

[98] Lee C., Kim B.G., Kim J.H., Chun J., Im J.P., Kim J.S. (2017). Sodium Butyrate Inhibits the NF-Kappa B Signaling Pathway and Histone Deacetylation, and Attenuates Experimental Colitis in an IL-10 Independent Manner. Int. Immunopharmacol..

[99] Bach Knudsen K.E., Lærke H.N., Hedemann M.S., Nielsen T.S., Ingerslev A.K., Gundelund Nielsen D.S., Theil P.K., Purup S., Hald S., Schioldan A.G. (2018). Impact of Diet-Modulated Butyrate Production on Intestinal Barrier Function and Inflammation. Nutrients.

[100] Saleem G.N., Gu R., Qu H., Bahar Khaskheli G., Rashid Rajput I., Qasim M., Chen X. (2024). Therapeutic Potential of Popular Fermented Dairy Products and Its Benefits on Human Health. Front. Nutr..

[101] Ward C.P., Perelman D., Durand L.R., Robinson J.L., Cunanan K.M., Sudakaran S., Sabetan R., Madrigal-Moeller M.J., Dant C., Sonnenburg E.D. (2025). Effects of Fermented and Fiber-Rich Foods on Maternal & Offspring Microbiome Study (FeFiFo-MOMS)—Study Design and Methods. Contemp. Clin. Trials.

[102] Wang Y., Rui B., Ze X., Liu Y., Yu D., Liu Y., Li Z., Xi Y., Ning X., Lei Z. (2024). Sialic Acid-Based Probiotic Intervention in Lactating Mothers Improves the Neonatal Gut Microbiota and Immune Responses by Regulating Sialylated Milk Oligosaccharide Synthesis via the Gut–Breast Axis. Gut Microbes.

[103] Wang C., Wei S., Liu B., Wang F., Lu Z., Jin M., Wang Y. (2022). Maternal Consumption of a Fermented Diet Protects Offspring against Intestinal Inflammation by Regulating the Gut Microbiota. Gut Microbes.

[104] Ortiz-Andrellucchi A., Sánchez-Villegas A., Rodríguez-Gallego C., Lemes A., Molero T., Soria A., Peña-Quintana L., Santana M., Ramírez O., García J. (2008). Immunomodulatory Effects of the Intake of Fermented Milk with *Lactobacillus casei* DN114001 in Lactating Mothers and Their Children. Br. J. Nutr..

[105] Wastyk H.C., Fragiadakis G.K., Perelman D., Dahan D., Merrill B.D., Yu F.B., Topf M., Gonzalez C.G., Van Treuren W., Han S. (2021). Gut-Microbiota-Targeted Diets Modulate Human Immune Status. Cell.

[106] Xi M., Yan Y., Duan S., Li T., Szeto I.M.-Y., Zhao A. (2024). Short-Chain Fatty Acids in Breast Milk and Their Relationship with the Infant Gut Microbiota. Front. Microbiol..

[107] Herías M.V., Cruz J.R., González-Cossío T., Nave F., Carlsson B., Hanson L.Å. (1993). The Effect of Caloric Supplementation on Selected Milk Protective Factors in Undernourished Guatemalan Mothers. Pediatr. Res..

[108] van den Elsen L.W.J., Garssen J., Burcelin R., Verhasselt V. (2019). Shaping the Gut Microbiota by Breastfeeding: The Gateway to Allergy Prevention?. Front. Pediatr..

[109] Al-Beltagi M. (2025). Human Milk Oligosaccharide Secretion Dynamics during Breastfeeding and Its Antimicrobial Role: A Systematic Review. World J. Clin. Pediatr..

[110] Fatimah, Massi M.N., Febriani A.D.B., Hatta M., Karuniawati A., Rauf S., Wahyuni S., Hamid F., Alasiry E., Patellongi I. (2022). The Role of Exclusive Breastfeeding on SIgA and Lactoferrin Levels in Toddlers Suffering from Acute Respiratory Infection: A Cross-Sectional Study. Ann. Med. Surg..

[111] Guerrant R.L., Schorling J.B., McAuliffe J.F., De Souza M.A. (1992). Diarrhea as a Cause and an Effect of Malnutrition: Diarrhea Prevents Catch-up Growth and Malnutrition Increases Diarrhea Frequency and Duration. Am. J. Trop. Med. Hyg..

[112] Baqui A., Sack R., Black R., Chowdhury H., Yunus M., Siddique A. (1993). Cell-Mediated Immune Deficiency and Malnutrition Are Independent Risk Factors for Persistent Diarrhea in Bangladeshi Children. Am. J. Clin. Nutr..

[113] Maares M., Keil C., Straubing S., Robbe-Masselot C., Haase H. (2020). Zinc Deficiency Disturbs Mucin Expression, O-Glycosylation and Secretion by Intestinal Goblet Cells. Int. J. Mol. Sci..

[114] Wan Y., Zhang B. (2022). The Impact of Zinc and Zinc Homeostasis on the Intestinal Mucosal Barrier and Intestinal Diseases. Biomolecules.

[115] Astiazarán-García H., Iñigo-Figueroa G., Quihui-Cota L., Anduro-Corona I. (2015). Crosstalk between Zinc Status and Giardia Infection: A New Approach. Nutrients.

[116] Thornton K.A., Mora-Plazas M., Marín C., Villamor E. (2014). Vitamin A Deficiency Is Associated with Gastrointestinal and Respiratory Morbidity in School-Age Children. J. Nutr..

[117] Mayo-Wilson E., Imdad A., Herzer K., Yakoob M.Y., Bhutta Z.A. (2011). Vitamin A Supplements for Preventing Mortality, Illness, and Blindness in Children Aged under 5: Systematic Review and Meta-Analysis. BMJ.

[118] Ullah I., Lang M. (2023). Key Players in the Regulation of Iron Homeostasis at the Host-Pathogen Interface. Front. Immunol..

[119] Hussein E.M., Zaki W.M., Ahmed S.A., Almatary A.M., Nemr N.I., Hussein A.M. (2016). Predominance of *Giardia lamblia* Assemblage A among Iron Deficiency Anaemic Pre-School Egyptian Children. Parasitol. Res..

[120] Qin Y., Shi W., Zhuang J., Liu Y., Tang L., Bu J., Sun J., Bei F. (2019). Variations in Melatonin Levels in Preterm and Term Human Breast Milk during the First Month after Delivery. Sci. Rep..

[121] Häusler S., Lanzinger E., Sams E., Fazelnia C., Allmer K., Binder C., Reiter R.J., Felder T.K. (2024). Melatonin in Human Breast Milk and Its Potential Role in Circadian Entrainment: A Nod towards Chrononutrition?. Nutrients.

[122] Rosen-Carole C.B., Greenman S., Wang H., Sonawane S., Misra R., O’Connor T., Järvinen K., D’Angio C., Young B.E. (2024). Association between Maternal Stress and Premature Milk Cortisol, Milk IgA, and Infant Health: A Cohort Study. Front. Nutr..

[123] Groer M., Davis M., Steele K. (2004). Associations between Human Milk SIgA and Maternal Immune, Infectious, Endocrine, and Stress Variables. J. Hum. Lact..

[124] Moirasgenti M., Doulougeri K., Panagopoulou E., Theodoridis T. (2019). Psychological Stress Reduces the Immunological Benefits of Breast Milk. Stress Heal.

[125] Tartibian B., Massart A. (2019). The Effect of Moderate Intensity Aerobic Exercise on Breast Milk IgA Concentrations. New Approaches Exerc. Physiol..

[126] Lovelady C.A., Hunter C.P., Geigerman C. (2003). Effect of Exercise on Immunologic Factors in Breast Milk. Pediatrics.

[127] Koivula T., Lempiäinen S., Rinne P., Hollmén M., Sundberg C.J., Rundqvist H., Minn H., Heinonen I. (2023). Acute Exercise Mobilizes CD8^+^ Cytotoxic T Cells and NK Cells in Lymphoma Patients. Front. Physiol..

[128] Koivula T., Lempiäinen S., Neuvonen J., Norha J., Hollmén M., Sundberg C.J., Rundqvist H., Minn H., Rinne P., Heinonen I. (2024). The Effect of Exercise and Disease Status on Mobilization of Anti-Tumorigenic and pro-Tumorigenic Immune Cells in Women with Breast Cancer. Front. Immunol..

